# A Rare Case of C2 Sensory Blockade with Preserved Phrenic Nerve Function in an Obstetric Patient

**DOI:** 10.1155/2016/3064373

**Published:** 2016-07-31

**Authors:** John C. Coffman, Kasey Fiorini, Meghan Cook, Robert H. Small

**Affiliations:** Department of Anesthesiology, The Ohio State University Wexner Medical Center, Columbus, OH 43210, USA

## Abstract

High neuraxial blockade is a serious complication in obstetric patients and requires prompt recognition and management in order to optimize patient outcomes. In cases of high neuroblockade, patients may present with significant hypotension, dyspnea, agitation, difficulty speaking or inability to speak, or even loss of consciousness. We report the unusual presentation of an obstetric patient that remained hemodynamically stable and had the preserved ability to initiate breaths despite sensory blockade up to C2. The presence of differential motor and sensory block documented in this case helped enable the patient to be managed with noninvasive ventilatory support until the high blockade regressed and we are not aware of any other similar reports in literature.

## 1. Introduction

High neuroblockade is an important cause of anesthesia-related maternal morbidity and mortality [1–3] and has been reported as the most common among serious complications in obstetric patients with an incidence of 1 in 4,336 neuraxial anesthetics [2]. We present an uncommon case of rapid epidural dosing for emergent cesarean delivery that led to sensory blockade up to the C2 dermatomal level, which remarkably did not result in hypotension, altered mentation, or loss of consciousness. The patient also retained at least partial diaphragmatic motor function despite the C2 sensory blockade, and this differential block along with her hemodynamic stability allowed her to be managed with mask-assisted respirations.

## 2. Case Presentation

A healthy 33-year-old nulliparous patient (weight = 94 kg, body mass index = 33.6 kg/m^2^) of 40 weeks' gestation presented in active labor and had uncomplicated epidural catheter placement at L3-4. Epidural catheter aspiration and test dose with 1.5% lidocaine with 1 : 200,000 epinephrine 3 mL were negative for signs or symptoms of intravascular or intrathecal catheter placement. Labor analgesia was initiated with 0.125% bupivacaine 10 mL and fentanyl 20 mcg, given in divided doses over five minutes, and this resulted in effective pain relief with a T10 sensory blockade to pinprick stimuli and partial motor blockade (Bromage score = 1). For the next three hours, she had good labor analgesia with a continuous epidural infusion of 0.0625% bupivacaine + 2 mcg/mL fentanyl at 12 mL/hr with patient-controlled epidural analgesia (6 mL bolus, 15 minute lockout). At this time, recurrent late fetal heart rate (FHR) decelerations became apparent, including a prolonged FHR deceleration to the 60–70 bpm range that resolved after a minute. The nonreassuring FHR pattern persisted despite placing the patient on supplemental oxygen and attempts at repositioning her in order to optimize uteroplacental perfusion, and at this point the obstetrician communicated the need for emergent cesarean delivery. The patient received oral sodium citrate 15 mL and was rapidly transitioned to the operating room. A T12 sensory block to pinprick stimuli and Bromage score of 0 were noted prior to epidural dosing with fentanyl 50 mcg and 2% lidocaine with 1 : 200,000 epinephrine 20 mL, given in 5 mL increments over five minutes' time. As the epidural was being dosed, a sterile surgical field was prepared and she was placed on nasal cannula oxygen 3 L/min given the nonreassuring fetal status. She was also given intravenous (IV) ephedrine 10 mg and glycopyrrolate 0.2 mg during this interval. The overnight anesthesia provider commonly administers these IV medications when dosing labor epidurals for cesarean delivery in order to minimize incidence of hypotension due to neuraxial blockade, though this is not the regular practice of other providers at our institution. Three minutes after epidural dosing, she was noted to have a T9-10 block to pinprick stimuli and almost complete motor block (Bromage score = 2). Epidural 3% 2-chloroprocaine 5 mL was administered to try and achieve a higher level for surgical anesthesia, and after one minute a T6-7 sensory level was noted immediately before skin incision.

The anesthesia staff began transitioning to the daytime shift as surgery was beginning. Delivery of a healthy female infant occurred two minutes after skin incision, and neonatal Apgar scores were 8 at one minute and 9 at five minutes. No intraoperative cause for the nonreassuring FHR tracing was identified. Just prior to delivery, the patient reported difficulty breathing and was immediately transitioned to hand-assisted ventilation through the anesthesia circuit and facemask. On further examination, she was completely unable to move her upper extremities or turn her head. However, she was able to speak quietly, initiate breaths, cough, blink her eyes, protrude her tongue, and move her facial muscles. She had sensory blockade at the C2 dermatome level, as she was unable to feel pinprick stimuli on her arms, neck, and posterior scalp areas. Her facial sensation remained intact. Repeat epidural catheter aspiration appeared negative for cerebrospinal fluid (CSF).

She did not display any hypotension, bradycardia, or loss of consciousness with the development of high neuroblockade. Her blood pressure ranged within 105–125/50–65 mm Hg and heart rate was 103–112 beats/min during the operative course, and she did not require additional vasopressor or inotropic or chronotropic support. She maintained a respiratory rate of 20–24 breaths/min and pulse oximetry 99-100% on mask-assisted ventilation with fraction of inspired oxygen of 50% and peak inspiratory pressure kept at 10–12 cm H_2_O using the adjustable pressure limiting valve. The mask-assisted ventilation, supplemental oxygen, and potentially the initial doses of IV ephedrine and glycopyrrolate allowed her to remain stable and comfortable. She did not desire intubation despite counseling regarding the potential risks of respiratory compromise due to muscle weakness and aspiration, though it was agreed that intubation would be performed if her clinical condition worsened. The willingness of the anesthesia providers and patient to continue with noninvasive ventilatory support was impacted by the patient's hemodynamic stability, normal mentation, ease of mask-assisted ventilation, preserved laryngeal motor function, and at least partially preserved diaphragmatic function. Additionally, she had not had oral food intake for more than twelve hours and no clear liquid intake for more than three hours. IV metoclopramide 10 mg was administered to increase gastric emptying. The patient remained remarkably calm despite the high neuroblockade that occurred, though she eventually began to have mild anxiety and received IV midazolam 2 mg for anxiolysis, given in divided doses five minutes apart. The midazolam was given incrementally to minimize the risk of oversedation and hypoventilation in this patient requiring mask-assisted respirations. Epidural morphine 2 mg is usually given for postcesarean analgesia at our institution, but in this case it was decided to withhold epidural morphine given the complication of high neuroblockade and the fact that an intrathecal catheter could not be completely ruled out.

Forty-five minutes after the 3% 2-chloroprocaine dose, the sensory blockade to pinprick had regressed to the C3-4 level. Respirations were assisted for a total of one hour, at which point testing revealed a C5-6 sensory level. She was then able to resume unassisted ventilation with supplemental oxygen administered by simple facemask at 5 L/min. After another hour, she had a T2-3 sensory level, demonstrated full grip strength, and was breathing comfortably on nasal cannula at 2 L/min. She was transferred to the recovery area in stable condition. After two hours in recovery, her sensory blockade had regressed to the L1 level and motor function was returning in her lower extremities. The remainder of her hospital stay was uncomplicated and she was discharged along with her baby on postpartum day three.

## 3. Discussion

The incidence of high neuraxial block requiring intubation or conversion to general anesthesia was recently reported to be 1 of 4,336 neuraxial anesthetics in obstetric patients [2]. High neuroblockade has been associated with unrecognized intrathecal administration of local anesthetic [2, 3] and with spinal anesthesia after failed epidural block [2, 4, 5] but can also occur with epidural catheter dosing [2, 3]. While an unrecognized intrathecal catheter cannot be completely ruled out in this case, it is very unlikely given the patient had a continuous epidural infusion for three hours without developing high neuroblockade and catheter aspiration remained negative for CSF. In addition, the volume of local anesthetic dosed for cesarean delivery likely would have resulted in a total spinal blockade if it were an intrathecal catheter. Rapid dosing of epidural medications under emergent circumstances most likely led to the high blockade observed. Epidural 2% lidocaine with 1 : 200,000 epinephrine has been observed to take ten minutes to achieve a surgical blockade for cesarean delivery [6, 7], and thus the onset of neuraxial blockade from the initial dosing had not fully taken effect prior to the epidural administration of 3% 2-chloroprocaine. Anesthesia providers may be pressed to rapidly provide extension of epidural blockade for cesarean anesthesia in emergent or urgent circumstances, but this case serves as an important example that epidural dosing should be performed gradually and incrementally in order to optimize patient safety [8].

IV ephedrine and glycopyrrolate administration during epidural dosing may have aided in preventing the onset of hypotension, bradycardia, or loss of consciousness that can occur with high neuroblockade of this magnitude. It should be noted that there is not good evidence to support routine administration of IV ephedrine and glycopyrrolate to prevent onset of hypotension or bradycardia induced by neuraxial blockade. Phenylephrine is considered the first-line vasopressor for routine management of spinal or epidural-induced hypotension in obstetric patients at our institution and many others given that it is more effective at preventing hypotension and is associated with less fetal acidosis compared to ephedrine [9]. When given prior to spinal anesthesia for cesarean delivery, glycopyrrolate has been shown to increase heart rate and cardiac output while lowering vasopressor dosing requirements, though it has not been shown to improve maternal or neonatal outcomes and is not routinely administered in many practices [10]. In cases of high neuroblockade accompanied by significant hypotension or bradycardia, IV epinephrine is likely a more appropriate vasopressor selection given its rapid onset and the direct adrenergic agonist can effectively manage bradycardia due to blockade of cardioaccelerator fibers or from diminished venous return [11].

The phrenic nerve arises from C3–5 before providing motor innervation to the diaphragm [12], and despite demonstrating a C2 sensory blockade our patient retained at least partial diaphragmatic function exhibited by the ability to initiate breaths. Differential nerve block in which sensory blockade is several levels higher than motor blockade may occur with both spinal and epidural anesthesia [13, 14]. Differential motor and sensory blockade can be observed in routine clinical practice if lower extremity motor function and sensory levels are closely evaluated during initiation of spinal or epidural anesthesia, though we are unaware of any previous reports of preserved phrenic nerve function despite C2 sensory blockade. The differential blockade observed in this case contributed to the patient's ability to remain stable with hand-assisted ventilation for approximately 60 minutes, and this approach avoided the need for intubation and general anesthesia.

Obstetric patients undergoing cesarean delivery are considered to be at elevated risk for pulmonary aspiration of gastric contents, and aspiration risk was an important consideration in the management of our patient. Aspiration accounted for 23% of anesthesia-related maternal deaths in the United States from 1979 to 1990, though a more recent report did not list aspiration as a cause of anesthesia-related mortality from 1990 to 2002 [1, 15]. A recent report of serious complications in obstetric anesthesia did not document any cases of aspiration in more than 257,000 anesthetics, including more than 5000 general anesthetics [2]. The decreasing incidence of significant aspiration events may be due to increased use of neuraxial anesthesia for cesarean delivery, adherence to* nil per os* standards for parturients, and use of aspiration prophylaxis medications. Based on these reports, it is apparent that maternal aspiration is rare in current practice, though it is difficult to counsel patients on the exact incidence of aspiration in obstetric patients. The patient in this case had not had recent oral intake and also received aspiration prophylaxis in the form of sodium citrate and metoclopramide in an attempt to reduce the likelihood or severity of a potential aspiration event. Importantly, she maintained the ability to cough and protect her airway from aspiration, which was expected even in the setting of sensory blockade up to C2 given that laryngeal innervation is through the recurrent laryngeal and superior laryngeal nerve branches of the vagus nerve [16]. Endotracheal intubation at the time high neuraxial blockade became apparent was considered as an option, but the anesthesia providers and patient felt comfortable continuing with mask-assisted ventilation in the setting of hemodynamic stability, normal mentation, preserved vocal cord function, no recent oral intake, and relative ease of mask-assisted breathing. Changes in any of these factors would likely have prompted endotracheal intubation, and thus it was important to have IV induction medications and intubating equipment immediately available.
